# Knowledge of modifiable risk factors of Coronary Atherosclerotic Heart Disease (CASHD) among a sample in India

**DOI:** 10.1186/1472-698X-9-2

**Published:** 2009-02-04

**Authors:** Omar Saeed, Vineet Gupta, Naveen Dhawan, Leanne Streja, John S Shin, Melvin Ku, Sanjeev Bhoi, Sanjay Verma

**Affiliations:** 1 Department of Medicine, Emory University, Atlanta, GA, USA; 2 Department of Emergency Medicine (JPNATC), All India Institute of Medical Sciences, (AIIMS), New Delhi, India; 3 Department of Medicine, University of California at Los Angeles, Los Angeles, CA, USA; 4 Department of Biostatistics, University of California at Los Angeles, Los Angeles, CA, USA; 5 Department of Medicine, University of California at Irvine, Irvine, CA, USA; 6 Department of Medicine, Michigan State University, East Lansing, MI, USA

## Abstract

**Background:**

The prevalence of Coronary Atherosclerotic Heart Disease (CASHD) is increasing in India. Several modifiable risk factors contribute directly to this disease burden. Public knowledge of such risk factors among the urban Indian population is largely unknown. This investigation attempts to quantify knowledge of modifiable risk factors of CASHD as sampled among an Indian population at a large metropolitan hospital.

**Methods:**

A hospital-based, cross sectional study was conducted at All India Institute of Medical Sciences (AIIMS), a major tertiary care hospital in New Delhi, India. Participants (n = 217) recruited from patient waiting areas in the emergency room were provided with standardized questionnaires to assess their knowledge of modifiable risk factors of CASHD. The risk factors specifically included smoking, hypertension, elevated cholesterol levels, diabetes mellitus and obesity. Identifying 3 or less risk factors was regarded as a poor knowledge level, whereas identifying 4 or more risk factors was regarded as a good knowledge level. A multiple logistic regression model was used to isolate independent demographic markers predictive of a participant's level of knowledge.

**Results:**

41% of the sample surveyed had a good level of knowledge. 68%, 72%, 73% and 57% of the population identified smoking, obesity, hypertension, and high cholesterol correctly, respectively. 30% identified diabetes mellitus as a modifiable risk factor of CASHD. In multiple logistic regression analysis independent demographic predictors of a good knowledge level with a statistically significant (p < 0.05) adjusted odds ratio (aOR) were: routine exercise of moderate intensity, aOR 8.41 (compared to infrequent or no exercise), no history of smoking, aOR 8.25, and former smokers, aOR 48.28 (compared to current smokers). Although statistically insignificant, a trend towards a good knowledge level was associated with higher levels of education.

**Conclusion:**

An Indian population in a hospital setting shows a lack of knowledge pertaining to modifiable risk factors of CASHD. By isolating demographic predictors of poor knowledge, such as current smokers and persons who do not exercise regularly, educational interventions can be effectively targeted and implemented as primary and secondary prevention strategies to reduce the burden of CASHD in India.

## Background

The high risk and wide prevalence of Coronary Atherosclerotic Heart Disease (CASHD) among the general Indian population is well established [1,2]. CASHD remains the highest cause of mortality in India, and the majority of cases are due to risk factors that include hypertension, smoking, Diabetes Mellitus (DM), and elevated serum cholesterol levels [3]. In particular, the incidence of acute myocardial infarction (AMI) in developing countries like India is especially alarming because it contributes to one third of all deaths stemming from heart disease. The gravity of this situation is emphasized by a recent projection from the WHO and the Indian Council of Medical Research (ICMR), which predicts that India will be the MI capital of the world by 2020. Sharma and Ganguly [4] call for urgency in reducing premature coronary artery disease risks and point to the prevention of CASHD before onset as a better suited method than intervening at a later stage of disease development. This has led members of the medical community to press for a concerted educational drive towards prevention of heart disease [5].

Knowledge of the predisposing risk factors is an important step in the modification of lifestyle behaviors conducive to optimal cardiovascular health in developing countries [6,7]. One method of targeting preventive educational strategies involves measuring and appropriately disseminating knowledge of the modifiable risk factors. However, the level of awareness of cardiovascular health modifiers among the Indian population has not been clearly quantified. A lack of cardiovascular health knowledge in the general population in neighboring Pakistan is demonstrated by a study in Karachi that reports limited knowledge of modifiable risk factors of heart disease in patients who had experienced an acute myocardial infarction [8,9]. This Pakistani study isolated specific demographic factors that correlate with lower knowledge of CASHD risk factors, such as fewer than 10 years of formal education, current usage of tobacco, and a nuclear family. A similar study in Saudi Arabia shows that physically inactive people were least aware of their risk of CASHD (10). Other investigations in the Western world, such as that of Potvin et.al. [11] in Canada show that individuals at greater risk of cardiovascular disease, such as the elderly and those with low education levels are least able to recall risk factors associated with CASHD. These studies set a precedence in finding specific demographic predictors for a poor level of knowledge of CASHD in a sample Indian population for effective targeting of preventive educational strategies.

The risk of CASHD is greater in urban settings compared to rural areas of India [12]. A particularly high prevalence of risk factors has been noted in industrial settings[13]. This warrants attention in assessing the knowledge of CASHD among those living in large cities, e.g. Mumbai and New Delhi. Yet, there are no measures of knowledge of modifiable risk factors of CASHD among the general Indian population in an urban center. Our study evaluated the knowledge levels of the modifiable risk factors among people who were present at a major tertiary hospital in New Delhi. The risk factors included smoking, hypertension, elevated cholesterol levels, DM, and obesity. We also identified gaps in the knowledge of specific risk factors as well as key demographic segments, with significantly poor levels of knowledge pertaining to modifiable risk factors of CASHD.

## Methods

### Study site

The All India Institute of Medical Sciences (AIIMS) in New Delhi was the chosen venue for the study, being one of the largest government funded research centers with a main hospital that receives hundreds of patients from all over India on a daily basis. The main hospital houses 1766 beds and has a staff of 1323 physicians along with 1810 nurses who treat a plethora of diseases and disorders. Nearly 400 patients arrive at AIIMS for emergency care daily, and the waiting area of this emergency facility was suitable for our survey.

### Participants

The emergency care waiting area is a separate covered hall where patients and their families stay during often lengthy periods of time. In this waiting area, members of the research team requested patients and their family members to fill out the survey, including demographic information (see table 1 and Additional file 1). Eligibility criteria for inclusion included their presence in the waiting area, an age of 18 years or older, and the ability to read or understand Hindi. A total of 217 subjects met these inclusion criteria and consented to the study. Our survey, methodology, and research proposal were reviewed and approved by the institutional review board of AIIMS. Prior to survey administration trained personnel on the research team obtained the informed consent from each participant.

**Table 1:** Demographic characteristics of participants.

Characteristics	Participants (%) n = 217
Age	
Mean (SD)	35.2 (SD: 10.44)
Gender	
Male	177 (82)
Female	40 (18)
Years of formal education	
None	0 (0)
Less than 5 years	10 (4.6)
6–10 years	27 (12.4)
Completed bachelors degree/Some college	152 (70)
Graduate school	28 (13)
History of smoking	
Current smoker	51 (23.5)
-Former smoker	60 (27.6)
-Never smoked	104 (48)
Exercise	
-None or infrequent	125 (57.6)
-Routinely *	92 (42.4)
Known diagnosis DM	
Yes	2 (1)
No	215 (99)
Known diagnosis Hypertension	
Yes	20 (9)
No	197 (91)
Known history of myocardial infarction	
Yes	15 (7)
No	197 (91)

*At least 3 times week, and greater than 30 minutes during each session

### Survey questionnaire and analytical design

The questionnaire used was originally prepared in English and consisted of knowledge-based questions assessing the participant's ability to identify correctly 5 modifiable risk factors of CASHD. This questionnaire (Additional file 1) was then translated into the Hindi language, and thoroughly checked by bilingual speakers to assure that questions intentionally worded as negatives, to make the true/false nature of correct answers vary, remained appropriately meaningful. Furthermore, individuals who spoke only Hindi verified the correct impression and idiomatic meaning of each question to the research team. Before filling out the survey, participants were instructed to only mark "yes" for risk factors that they felt definitely contribute to CASHD, otherwise to mark "not sure." The research team emphasized that discussion of responses should not occur between subjects and this was enforced by direct observation of the participants during completion of the survey.

Out of the ten risk factors listed in the survey, five are clearly known to cause CASHD. These modifiable risk factors are smoking, hypertension, high cholesterol levels, DM and obesity. Additional non-associated risk factors were included in the questionnaire to maintain validity of responses and avoid false positives that could be generated by random responses. After participants completed the questionnaires, the survey forms were collected and the correct answers were explained.

**Figure 1:**
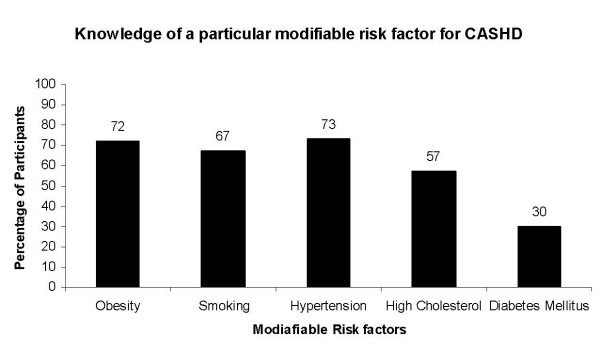
**Percentage of participants (n = 217) with knowledge of a specific risk factor for Coronary Atherosclerotic Heart Disease (CASHD)**.

Identifying three or less risk factors was regarded as a poor level of knowledge, while correctly knowing four or more was regarded as a good level of knowledge. This grouping of knowledge levels was based on another study that had assessed knowledge of heart disease [7]. Demographics of participants and their responses are shown in table 1 and figure 1.

### Statistical Analysis

Descriptive analyses were used to assess the distribution of our data. Univariate logistic regression generated crude odds ratios to study the relationship of each independent demographic variable to the outcome of "good" *versus *"poor" levels of knowledge. A multiple logistic regression model was created to isolate statistically significant demographic predictors associated with a "good" level of knowledge. This multiple regression analysis yielded adjusted odds ratios after controlling for the following confounding variables of knowledge: age, gender, and known medical history of hypertension, DM and myocardial Infraction. Crude and adjusted odds ratios with 95% CI are given in table 2. All statistical analysis was conducted with SAS software, version 9.0.

**Table 2:** Crude and adjusted odds ratios associating a "good" level of knowledge of modifiable risk factors with specific demographic variables

Demographic	Crude odds ratio	95% CI	*Adjusted odds ratio	95% CI
Age				
-Less than or equal to 35	1.00	-	confounding variable	
-Greater than 35	0.59	(0.34–1.02)		
				
Gender				
-Male	1.00	-	confounding variable	
-Female	0.816	(0.40–1.65)		
				
Exercise				
-No	1.00	-	1.00	-
-Yes**	6.42	(3.48–11.28)	8.41**	(4.29–16.46)
				
Level of formal education received				
-Less than or equal to 5 years	1.00	-	1.00	-
-6–10 years	0.82	(0.16–4.01)	0.91	(0.16–5.01)
-Bachelors or some college	3.36	(0.79–14.26)	3.64	(0.83–16.01)
-Graduate school	3.61	(0.77–17.00)	3.98	(0.83–19.29)
				
History of Smoking cigarettes				
-Current smoker	1.00	-	1.00	-
-Former smoker**	38.6	(11.83–126.04)	48.28**	(13.9–167.58)
-Never smoked**	7.34	(2.4–21.94)	8.25**	(2.53–26.72)
				
Known diagnosis of Hypertension				
-No	1.00	-	confounding variable	
-Yes	1.46	(0.58–3.67)		
				
Known diagnosis of DM				
-No	1.00	-	confounding variable	
-Yes	1.42	(0.087–22.94)		
				
Known history of Myocardial Infarction				
-No	1.00	-	Confounding variable	
-Yes	0.49	(0.15–1.60)		

*Adjusted for confounding variables of knowledge such as age, gender, and known medical history of hypertension, DM and myocardial infarction.

** Demographic variables significantly associated with a "good" level of knowledge

## Results

The mean age of the 217 participants was 35, of which 82% were males. About 70% of the participants had completed a bachelor's degree or attended some college, while only 4.6% completed less than five years of formal education. About 48% of the participants had never smoked, while 27.5% were former smokers and 23.5% currently smoked. Routine exercise of moderate intensity was undertaken by 42%. In assessing past medical history, only 1% reported a known history of DM, and 9% and 7% had known histories of hypertension and myocardial infarction, respectively (see Table 1).

Of the 217 participants, smoking, obesity, hypertension, and high cholesterol were identified as modifiable risk factors of CASHD by 68%, 72%, 73%, and 57%, respectively. Only 30% knew that DM is a modifiable risk factor of CASHD (figure 1). Overall, a majority of participants lacked the predefined "good" level of knowledge pertaining to modifiable risk factors of CASHD. Specifically, only 41.4% of the participants had a "good" level of knowledge *versus *58.6% showing a "poor" level.

Table 2 gives the crude and adjusted odds ratios of the participants' knowledge of modifiable risk factors of CASHD. We found that predictors of a good level of knowledge with a statistically significant aOR (p < 0.05) were as follows: 1) routine exercise of moderate intensity, aOR 8.41, 95% CI 4.29–16.46 versus infrequent or no exercise:, 2) no history of smoking, aOR 8.25, 95% CI 2.53–26.72: or 3) a former history of smoking, aOR 48.28, 95% CI 13.90–167.58 compared to current smokers. Although statistically insignificant, a trend towards a good knowledge level was associated with higher levels of education. These predictors were adjusted for internal confounders of knowledge such as age, gender, and known medical history of hypertension, DM or myocardial infraction.

## Discussion

The results indicate that the majority (58%) of individuals sampled lacked adequate awareness of modifiable risk factors of CASHD. On further analysis, knowledge gaps were evident as more participants were able to identify certain risk factors in comparison to others.

One particular area of interest is knowledge of cigarette smoking as a modifiable risk factor for CASHD. Importantly, 67.7% of participants correctly identified smoking cigarettes as a modifiable risk factor of CASHD. Damage due to smoking even surpasses CASHD as recent evidence suggests that nearly 20% of male premature deaths in India are related to it [14]. The importance of stopping smoking is emphasized by several health awareness campaigns in India and the introduction of laws banning smoking in public places by the Indian Government [15]. Those participants who did not smoke or were former smokers demonstrated significantly higher knowledge levels of CASHD risk factors than those currently smoking. Thus, the results indicate that specifically educating current smokers about the linkage between smoking and CASHD would have an optimal preventive impact.

A significant percentage (70%) of participants failed to identify DM as a risk factor. Our results are consistent with previous studies conducted among urban adult Indians that found a low level of awareness of DM [16,17]. Moreover, our study shows a knowledge gap in associating DM with CASHD among the Indian public. This finding is especially worrisome given that India is likely to experience the biggest absolute increase in the number of people with DM by the year 2030 [18]. Unfortunately, despite such a high predicted incidence, India lacks the infrastructure of educational facilities and programs vital to raising awareness and knowledge of DM and its contribution to atherosclerotic cardiovascular disease [16].

Our data notably reveals certain characteristics that are significant predictors of poor knowledge levels of modifiable risk factors. Participants who reported low levels of routine exercise and who are current smokers had a significantly poor knowledge level. This correlation between sub-optimal lifestyle practices and low knowledge levels is consistent with other studies from the nearby countries of Pakistan and Saudi Arabia, as mentioned above. This significant finding is important for public health officials and Indian health professionals to consider when evaluating patients and discussing prevention strategies.

There are several notable limitations to our study. First, some patients who visited AIIMS for treatment came from rural areas near the outskirts of the large urban city of New Delhi, and from various parts of the country. Thus, our sample is not entirely comprised of an urban population. Yet, while the development of CASHD is more common in urban than rural areas, studies have suggested that there are increasing risk factors for CASHD and growing prevalence of vascular disease in rural areas [17], indicating the need for educational interventions for rural populations. Second, the survey questionnaire used was not rigorously validated prior to administration. However, it is valid since each of the correctly identifiable risk factors is proven to cause CASHD. Moreover, each risk factor serves as an independent knowledge domain because its identification is not influenced by other questions. A third limitation is that our sample population was relatively small with a high degree of variability in their responses, as evident by several large confidence intervals. Also, the demographics of the study participants might not be reflective of the entire Indian population; for example, the sample was predominantly male and generally of a higher level of education than the rest of India. Thus, any extrapolation of the findings to the entire population must take these limitations into account.

## Conclusion

Public awareness of risk factors for CASHD is essential, but no previous measures of it exist in India, where it is on the rise and the incidence of MI is dramatically increasing. Our study suggests that there is a lack of awareness among a sampled Indian population regarding modifiable risk factors of CASHD, especially DM. Educational interventions are needed to make the Indian public aware of modifiable risk factors of CASHD, and should specifically target individuals who do not exercise, currently smoke, and have less formal education to be optimally effective as a preventative measure.

## Competing interests

The authors declare that they have no competing interests.

## Authors' contributions

OS initially conceived the concept of measuring knowledge levels of CASHD in an Indian population and was involved with all aspects of this study. ND, VG, JS and MK were involved in writing and revising the manuscript. LS compiled data and provided statistical analysis. VG, SB and SV were part of the AIIMS research team in India that conducted the entire survey and assisted with completion of this investigation. All authors approved the final manuscript.

## Pre-publication history

The pre-publication history for this paper can be accessed here:

http://www.biomedcentral.com/1472-698X/9/2/prepub

## Supplementary Material

Additional file 1 **Appendix 1.** Survey given to participants. Click here for file
