# Supplementary material for: Knowledge of modifiable risk factors of Coronary Atherosclerotic Heart Disease (CASHD) among a sample in India
Source: BMC Int Health Hum Rights. 2009 Feb 4;9:2. doi: 10.1186/1472-698X-9-2 (PMC2642756; doi:10.1186/1472-698X-9-2)
Supplement: Additional file 1 — Appendix 1. Survey given to participants. [file 1472-698X-9-2-S1.doc]

# Appendix 1:Survey given to participants.

*Which of the following increases the risk of having a heart attack?*

| ***Question***  ***Number*** |  | ***Yes*** | ***No*** | ***Not sure*** |
| --- | --- | --- | --- | --- |
| 1 | Smoking cigarettes |  |  |  |
| 2 | Weight loss |  |  |  |
| 3 | Obesity |  |  |  |
| 4 | Depression |  |  |  |
| 5 | Hypertension |  |  |  |
| 6 | High cholesterol level in the blood |  |  |  |
| 7 | Daily exercise |  |  |  |
| 8 | Stress |  |  |  |
| 9 | Sleeping too much |  |  |  |
| 10 | Diabetes Mellitus |  |  |  |
